# Analysis of the molecular networks involved in primary cilium formation

**DOI:** 10.1186/2046-2530-1-S1-P15

**Published:** 2012-11-16

**Authors:** R Bonavita, A Luini, A Luini, A Colanzi

**Affiliations:** 1 Istituto di Biochimica delle Proteine, Italian National Research Council (CNR), Italy; 2 Telethon Institute of Genetics and Medicine (TIGEM), Italy

## 

To identify functional interactions among some important ciliopathy genes, we carried out searches related to genes representative of important ciliopathies, such as Bardet-Biedl syndrome (BBS), nephronophthisis, and oro-facial-digital syndrome type 1. We built a functional interaction network for the identification of conserved components that might regulate ciliary trafficking, and thus provide insight into the mechanisms of polycystic kidney disease. By mining the published protein-protein interaction data, we identified an interesting association between the exocyst complex (involved in targeting of post-Golgi vesicles to the plasma membrane), the BBSome (a multisubunit protein complex involved in the transport of cargo to the cilium), centriolar satellites, the centrosome and the Golgi complex. In addition, this analysis led us to the identification of a new centrosomal protein of previously undefined function involved in cilium formation. We are now investigating the molecular function of this protein, as it interacts physically and functionally with OFD1, a gene associated with ciliopathies.

